# Prevalence, Virulence Determinants, and Antimicrobial Resistance of *Staphylococcus aureus* and *Escherichia coli* Isolated from Computer Devices Used by Staff and Students at a Northern Thailand University

**DOI:** 10.3390/pathogens15030274

**Published:** 2026-03-03

**Authors:** Sorawit Upakut, Achiraya Siriphap, Ornampai Japa, Pathumwan Watsing, Peerapat Bunpak, Aacharaporn Ta-In, Cholthicha Inmanee, Chutamas Thepmalee, Nittiya Suwannasom, Krissana Khoothiam

**Affiliations:** 1 Division of Microbiology, School of Medical Sciences, University of Phayao, Phayao 56000, Thailand; sorawit.up@up.ac.th (S.U.); achiraya.si@up.ac.th (A.S.); ornampai.ja@up.ac.th (O.J.); pathumwan.wt@gmail.com (P.W.); peerapatbunpak@gmail.com (P.B.); aacharaporn.tn@gmail.com (A.T.-I.); cholthicha.cch@gmail.com (C.I.); 2 Division of Biochemistry, School of Medical Sciences, University of Phayao, Phayao 56000, Thailand; chutamas.th@up.ac.th (C.T.); nittiya.su@up.ac.th (N.S.)

**Keywords:** prevalence, Staphylococcus aureus, *Escherichia coli*, virulence gene, antimicrobial-resistant, computer device

## Abstract

Computer devices in university settings are frequently shared and repeatedly handled, making them potential reservoirs for pathogenic bacteria. This study aimed to investigate the prevalence, virulence determinants, and antimicrobial resistance profiles of *Staphylococcus aureus* and *Escherichia coli* isolated from computer devices used by staff and students at a university in Northern Thailand. A total of 400 computer devices were sampled, with each device defined as a single sampling unit comprising both the keyboard and computer mouse. Bacterial identification was performed using PCR, while staphylococcal enterotoxin (*se*) genes and diarrheagenic *E. coli* (DEC)-associated virulence genes were detected by PCR. Antimicrobial susceptibility was assessed using the disk diffusion method. Overall, 74 (18.5%) *S. aureus* isolates and 6 (1.5%) *E. coli* isolates were recovered. The highest prevalence of *S. aureus* was observed among personal-use student computer devices (29%; *p* < 0.001), whereas *E. coli* was most frequently detected on public-use staff computer devices (4%). Among *S. aureus* isolates, 24.3% (18/74) carried at least one *se* gene, with sec being the most prevalent (13.5%). Half of the *E. coli* isolates harbored the *astA* gene. Low resistance rates (<10%) were observed among *S. aureus*; however, four isolates (5.4%) were classified as MRSA, three of which exhibited multidrug resistance. All *E. coli* isolates were resistant to ampicillin, and 50% displayed multidrug-resistant phenotypes. These findings suggest that computer devices can act as occasional reservoirs of potentially pathogenic and antimicrobial-resistant bacteria in university environments.

## 1. Introduction

In the present era, electronic devices, particularly computers, have become integral to daily life worldwide, serving multiple purposes across professional, educational, and social contexts [1]. Within academic environments such as universities, computers are indispensable for students and staff, supporting activities including learning, teaching, research, communication, and academic administration [2]. As a result, computer peripherals—especially keyboards and computer mice—are subject to frequent and prolonged handling by multiple users. Despite their extensive use, these high-touch surfaces are often neglected during routine cleaning and disinfection, allowing them to function as potential reservoirs for microbial contamination and increasing the risk of pathogen transmission in educational settings [3].

Among the microorganisms reported on computer surfaces, *Staphylococcus aureus* and *Escherichia coli* are of particular concern due to their clinical and public-health relevance [4,5]. *S. aureus*, a Gram-positive coccus commonly found as part of the normal skin microbiota, is a well-recognized cause of skin and soft tissue infections and may lead to severe invasive diseases [4]. Moreover, strains producing staphylococcal enterotoxins pose a risk for foodborne intoxication, highlighting their importance in hygiene and sanitation surveillance [6]. The frequent detection of *S. aureus* on repeatedly touched surfaces raises concerns regarding its persistence and transmission in shared environments, particularly in light of the increasing global incidence of methicillin-resistant *S. aureus* (MRSA) [7]. In contrast, *E. coli* is a Gram-negative bacillus that inhabits the human intestinal tract and is widely used as an indicator of fecal contamination and sanitation status [8]. Certain pathogenic *E. coli* strains carry diarrheagenic *E. coli* (DEC)-associated virulence genes and are responsible for gastrointestinal diseases worldwide, with growing reports of multidrug-resistant strains further complicating infection control and treatment [9].

Antimicrobial resistance (AMR) has emerged as a critical global health challenge, significantly limiting therapeutic options for bacterial infections and increasing morbidity and mortality [10]. Surveillance of AMR in community and institutional environments is therefore essential to inform appropriate antimicrobial use and infection-prevention strategies [11]. Several studies have reported bacterial contamination and associated AMR profiles on computer keyboards and mice in different countries, demonstrating considerable variation in prevalence and resistance patterns [1,12,13]. However, data regarding the prevalence, virulence determinants, and antimicrobial resistance of *S. aureus* and *E. coli* on computer devices in universities in Northern Thailand remain limited. Therefore, the present study aimed to investigate the prevalence and antimicrobial resistance profiles of *S. aureus* and *E. coli* isolated from computer device used by staff and students at a university in Phayao Province, Northern Thailand. In addition, the study evaluated the distribution of staphylococcal enterotoxin (*se*) genes and DEC-associated virulence genes to provide insight into the potential public-health significance of bacterial contamination on frequently used computer devices.

## 2. Materials and Methods

### 2.1. Study Location and Sample Classification

This cross-sectional study was conducted from 1 March to 30 April 2025 at the University of Phayao, Muang District, Phayao Province, Northern Thailand. Surface parts of computer keyboards and computer mice were selected as sampling sites and sampled from devices used by staff and students at the university. The samples were categorized into four groups: public-use staff computers, personal-use staff computers, public-use student computers, and personal-use student computers. Public-use staff computers referred to computers located in university classrooms used for teaching, which are accessible to multiple staff members. Personal-use staff computers referred to computers used by individual staff members and located in personal offices or rooms. Public-use student computers referred to computers that could be accessed by many students, such as those in computer laboratories for information technology learning and computers located in the university library. Personal-use student computers referred to personal computers owned and used by individual students at the University of Phayao.

### 2.2. Sample Size Calculation

In this study, one computer (staff or student), consisting of a computer keyboard and a computer mouse, was considered as one sample. The total sample size was estimated using the standard formula for prevalence studies:

*n* = *Z*^2^ _1−*α*/2_
*P*(1 − *P*)/*e*^2^

where *n* is the required sample size, *Z*_1−*α*/2_ is the standard normal deviate corresponding to the desired confidence level, *P* is the expected prevalence, and *e* is the acceptable margin of error. Because prior prevalence data for *S. aureus* and *E. coli* carriage in computer keyboards and computer mice in the study area were unavailable, a conservative value of *P* = 0.50 was applied to maximize the required sample size. Based on this calculation, the estimated sample size was *n* = 385 computers. However, a total of 400 computers were collected in this study. The samples were equally distributed into four groups: public-use staff computers, personal-use staff computers, public-use student computers, and personal-use student computers, with 100 samples in each group.

### 2.3. Sample Collection

Samples were collected using sterile cotton swabs under aseptic conditions, as previously described with slight modifications [12]. Sample collection was carried out between 12:00 and 14:00 to ensure that the computer keyboards and computer mice had been touched by users prior to sampling. Each cotton swab was moistened by dipping it into 0.85% NaCl solution and then used to swab the surfaces of computer keyboards and mice. For keyboards, the moistened cotton swab was applied across all keys commonly used for typing, including letter keys, space bar, enter key, function keys, number keys, and other frequently touched keys. For computer mice, the cotton swab was applied to the palm rest area as well as the left and right click buttons. After sampling, the contaminated cotton swab was immediately transferred into a sterile test tube containing 2 mL of peptone water (PW) medium (HiMedia Laboratories Pvt. Ltd., Mumbai, India), vortexed, and incubated at 37 °C for 16–24 h.

### 2.4. Isolation of S. aureus and E. coli

After incubation, the cultured PW medium was transferred using a sterile loop and streaked onto selective agar media. Mannitol salt agar (MSA; HiMedia Laboratories Pvt. Ltd., Mumbai, India) was used for the isolation of *S. aureus*, while eosin methylene blue agar (EMB; HiMedia Laboratories Pvt. Ltd., Mumbai, India) was used for the isolation of *E. coli*. The inoculated plates were incubated at 37 °C for 24 h. Presumptive *S. aureus* colonies showing yellow coloration on MSA were selected. Presumptive *E. coli* colonies exhibiting a metallic sheen with a dark center on EMB agar were also selected for further examination. The selected colonies were subjected to morphological and biochemical characterization. Gram staining was performed, in which *S. aureus* appeared as Gram-positive cocci in grape-like clusters, while *E. coli* appeared as Gram-negative rods. Biochemical tests for *S. aureus* included oxidase, catalase, and coagulase tests [14]. Biochemical tests for *E. coli* included sugar fermentation, methyl red, indole, and citrate tests [15]. Confirmed colonies of *S. aureus* and *E. coli* were subcultured and maintained on nutrient agar (NA) medium (HiMedia Laboratories Pvt. Ltd., Mumbai, India) and incubated at 37 °C for 24 h for further analysis.

### 2.5. Molecular Confirmation of S. aureus and E. coli

Presumptive *S. aureus* and *E. coli* isolates were confirmed using the PCR method. Bacterial DNA was extracted by the boiling method as previously described [16]. Briefly, a single colony grown on nutrient agar (NA) medium was picked into a test tube containing 100 μL of sterile distilled water and heated at 100 °C using a heat block. The tube was then cooled on ice for 5 min and centrifuged at 10,000 rpm for 10 min. The supernatant was transferred into a new sterile tube and used as the DNA template for PCR. Species-specific genes were used for molecular identification, including the *femA* gene for *S. aureus* [17] and the *uidA* gene for *E. coli* [18]. PCR amplification was performed as previously described in our studies [19,20]. Single PCR amplification was performed for each target gene, and multiplex PCR was not applied in this study. PCR detection was performed using previously validated primers and confirmed positive control strains described in earlier studies [19,21]. Briefly, single PCR reactions were carried out in a total volume of 25 μL following the OnePCR™ Ultra protocol (Bio-Helix Co., Ltd., Keelung, Taiwan). The PCR mixture consisted of 12.5 μL of OnePCR™ Ultra master mix (containing Taq DNA polymerase, PCR buffer, dNTPs, gel loading dyes, enhancer, and fluorescence dye), 0.5 μM of species-specific primers (*femA*-F/*femA*-R for *S. aureus* or *uidA*-F/*uidA*-R for *E. coli*, as shown in Table 1), 1 μL of DNA template, and 10.5 μL of sterile distilled water. PCR conditions were performed according to previously described protocols [17,18]. In addition, oxacillin-resistant *S. aureus* isolates were further evaluated for the presence of methicillin resistance genes (*mecA* and *mecC*) to confirm MRSA status (OXA-resistant, MIC > 4 μg/mL), using primer sets (*mecA*-F/*mecA*-R and *mecC*-F/mecC-R) as shown in Table 1, and PCR conditions as previously described by Stegger et al. [22]. The PCR products were separated by 1.5% (*w*/*v*) agarose gel electrophoresis and visualized under ultraviolet light using a gel documentation system (BIS 303 PC, DNR Bio-Imaging Systems Ltd., Modi’in-Maccabim-Re’ut, Israel). Amplicons of the expected sizes (300 bp for *S. aureus femA*, 166 bp for *E. coli uidA*, 533 bp for *mecA*, and 304 bp for *mecC*) were subsequently purified and sequenced by a commercial sequencing service (Macrogen Inc., Seoul, Republic of Korea). The obtained nucleotide sequences were analyzed for similarity using the BLASTn program available at the NCBI GenBank database (National Center for Biotechnology Information, Bethesda, MD, USA; https://blast.ncbi.nlm.nih.gov, accessed on 3 February 2026).

### 2.6. Molecular Detection of Virulence Genes

All *S. aureus* isolates were evaluated for nine *se* genes, including *sea*, *seb*, *sec*, *sed*, *sej*, *ser*, *sem*, *sel*, and *seq*. *E. coli* isolates were evaluated for ten DEC-associated virulence genes, including *aggR*, *stx1*, *stx2*, *astA*, *estp*, *esth*, *elt*, *bfpA*, *eae*, and *invE*, using PCR. PCR assays were performed using gene-specific primers, as shown in Table 1. PCR amplification was carried out according to the protocols previously described in our studies [19,21].

### 2.7. Antimicrobial Susceptibility Testing

Antimicrobial susceptibility testing of all *S. aureus* and *E. coli* isolates was performed using the disk diffusion method in accordance with the guidelines of the Clinical and Laboratory Standards Institute (CLSI) [23]. For *S. aureus*, a total of 16 antimicrobial agents were tested, including gentamicin, penicillin G, oxacillin, cefoxitin, erythromycin, vancomycin, chloramphenicol, ciprofloxacin, levofloxacin, moxifloxacin, clindamycin, linezolid, tigecycline, fusidic acid, novobiocin, and trimethoprim/sulfamethoxazole. For *E. coli*, susceptibility testing was carried out using 15 antimicrobial agents, including gentamicin, streptomycin, kanamycin, ampicillin, amoxicillin/clavulanic acid, imipenem, meropenem, cefotaxime, cefoxitin, tetracycline, ceftazidime, tigecycline, chloramphenicol, erythromycin, and trimethoprim/sulfamethoxazole. Mueller–Hinton agar (HiMedia Laboratories Pvt. Ltd., Mumbai, India) was used as the test medium for the disk diffusion assay. *S. aureus* TISTR 746 and *E. coli* TISTR 073 were included as reference strains for quality control. MDR was defined as resistance to one or more antimicrobial agents in three or more different antimicrobial classes, according to established criteria [24].

### 2.8. Confirmation of Oxacillin and Vancomycin Resistance by Broth Microdilution MIC Method

*S. aureus* isolates previously identified as oxacillin- and vancomycin-resistant by the disk diffusion method were confirmed by the broth microdilution minimum inhibitory concentration (MIC) method according to CLSI guidelines [13]. Briefly, a single bacterial colony was adjusted to a 0.5 McFarland turbidity standard in sterile water. The bacterial suspension was subsequently diluted in Mueller–Hinton broth (MHB; HiMedia Laboratories Pvt. Ltd., Mumbai, India) to obtain the recommended final inoculum density for broth microdilution testing. Serial two-fold dilutions of oxacillin and vancomycin were prepared in sterile 96-well microplates containing MHB medium. The inoculated microplates were incubated aerobically at 35–37 °C for 18–24 h, after which MIC values were determined as the lowest antimicrobial concentration showing complete inhibition of visible bacterial growth. Results were interpreted according to current CLSI breakpoints for staphylococci.

### 2.9. Statistical Analysis

The prevalence rates of *S. aureus* and *E. coli* recovered from computer devices, as well as virulence profiles, and AMR among the four sample groups (public-use staff, personal-use staff, public-use students, and personal-use students), were presented as percentages. Comparisons of *S. aureus* and *E. coli* prevalence, and virulence-gene positivity among the four groups were assessed using the chi-square test. Statistical analyses were performed using GraphPad Prism software (version 5.00 for Windows), and *p*-values < 0.05 were considered statistically significant.

## 3. Results

### 3.1. Prevalence of S. aureus and E. coli Isolates

In this study, a total of 74 (18.5%) *S. aureus* isolates and 6 (1.5%) *E. coli* isolates were recovered from 400 computer device samples used by staff and students at a university in Northern Thailand. The highest prevalence of *S. aureus* was observed in the personal-use student computer group (29%), followed by the personal-use staff computer group (21%), the public-use staff computer group (20%), and the public-use student computer group (4%), respectively (Table 2). A statistically significant difference in *S. aureus* prevalence was observed among the different computer-use groups (*p* < 0.0001). Regarding *E. coli*, the highest prevalence was detected in public-use staff computers (4%), followed by public-use student computers and personal-use student computers, both at 1% (Table 2). No *E. coli* isolates were recovered from the personal-use staff computer group. No statistically significant difference was observed in *E. coli* prevalence among the four groups.

### 3.2. Distribution of se Genes in S. aureus and DEC-Associated Virulence Genes in E. coli Isolates

A total of 74 *S. aureus* isolates were screened for nine *se* genes. The results showed that 18 (24.3%) *S. aureus* isolates carried at least one *se* gene, as shown in Table 3. The highest prevalence of *se*-gene-positive isolates was observed in the personal-use staff computer group (38.1%), followed by the public-use student computer group (25%), the personal-use student computer group (24.1%), and the public-use staff computer group (10%), respectively. No statistically significant difference in the distribution of *se*-gene-positive *S. aureus* isolates was observed among the sample groups (*p* = 0.2219). The most prevalent *se* gene detected was *sec* (13.5%; 10/74), followed by *sea* (5.4%; 4/74), *sed* and *sem* (both 2.7%; 2/74), and *seq* (1.4%; 1/74). However, *seb*, *sej*, *sel*, and *ser* were not detected. In addition, this study identified one *S. aureus* isolate carrying a two-gene se profile (*sea*–*sed*), which was recovered from the personal-use staff computer group.

Six *E. coli* isolates were evaluated for the presence of ten DEC-associated virulence genes. The results showed that 3 (50%) of these *E. coli* isolates harbored at least one virulence gene, as indicated in Table 4. Only one virulence gene profile, *astA*, was detected. This gene was identified in two *E. coli* isolates recovered from the public-use staff computer group and in one *E. coli* isolate recovered from the public-use student computer group. No statistically significant difference was observed in the distribution of virulence-gene-positive *E. coli* isolates among the computer-use groups (*p* = 0.3149).

### 3.3. Antimicrobial Resistance Phenotypes of S. aureus and E. coli Isolates

**Figure 1:**
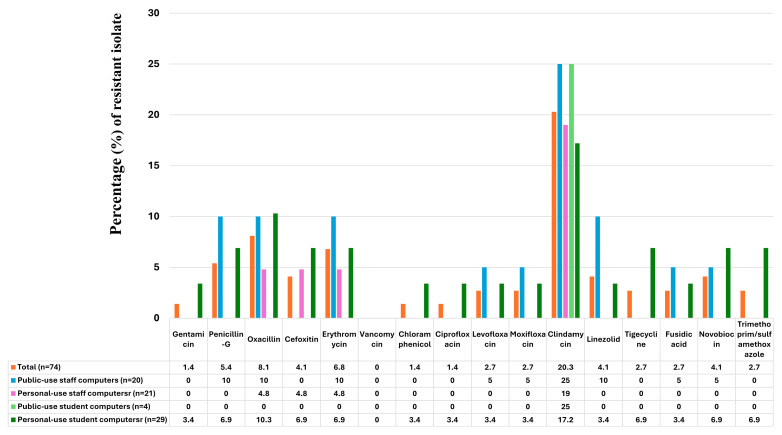
Antibiotic resistance profiles of *Staphylococcus aureus* isolates obtained from public- and personal-use staff and student computer groups.

The present study describes the antimicrobial resistance profiles of *S. aureus* isolates recovered from different computer-use groups, based on susceptibility testing against 16 antimicrobial agents. Among the 74 *S. aureus* isolates, the highest resistance rate was observed for clindamycin (20.3%) as indicated in Figure 1. Most *S. aureus* isolates exhibited low resistance frequencies (<10%) to the remaining antimicrobial agents, including oxacillin (8.1%), erythromycin (6.8%), penicillin G (5.4%), and cefoxitin (4.1%). Resistance to vancomycin, linezolid, and novobiocin was also observed at 4.1%. Lower resistance rates were detected for levofloxacin, moxifloxacin, tigecycline, fusidic acid, and trimethoprim/sulfamethoxazole (2.7%), as well as gentamicin, chloramphenicol, and ciprofloxacin (1.4%). Additionally, six *S. aureus* isolates exhibited an oxacillin-resistant profile by disk diffusion; however, only four isolates were confirmed as oxacillin-resistant based on broth microdilution MIC values (MIC > 4 µg/mL). Three *S. aureus* isolates initially showed reduced susceptibility to vancomycin in disk diffusion screening; however, subsequent broth microdilution testing demonstrated MIC values within the susceptible range according to CLSI criteria. Therefore, no vancomycin-resistant *S. aureus* phenotype was identified.

**Figure 2:**
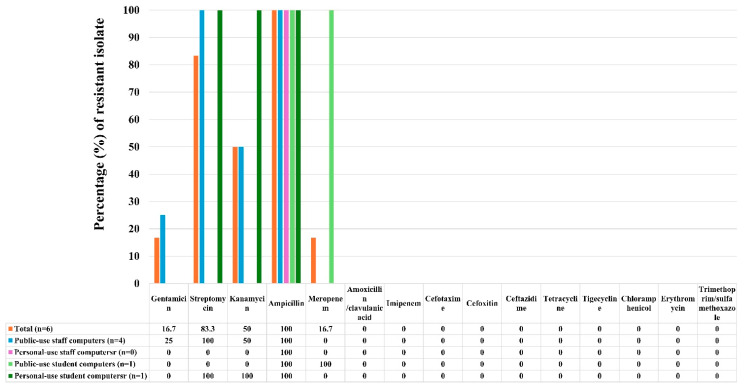
Antibiotic resistance profiles of *Escherichia coli* isolates obtained from public- and personal-use staff and student computer groups.

For *E. coli*, antimicrobial resistance profiles were evaluated using 15 antimicrobial agents. The results showed that all 6 *E. coli* isolates were resistant to ampicillin (100%) (Figure 2). High resistance was also observed for streptomycin (83.3%), followed by kanamycin (50%), and gentamicin and meropenem (both 16.7%). Notably, all *E. coli* isolates were susceptible to the remaining antimicrobial agents tested.

### 3.4. Association of Antimicrobial Resistance Patterns with se Genes in S. aureus

In this study, 29.7% (22/74) of *S. aureus* isolates exhibited resistance to at least one of the tested antimicrobial agents. Among these resistant isolates, 8.1% (6/74) were positive for *se* genes, with *sec* being the most frequently detected gene (4 isolates), as shown in Table 5. A total of 13 distinct AMR patterns were identified. The most prevalent AMR pattern was clindamycin resistance (CLI), which was detected in nine *S. aureus* isolates. Among these nine isolates, three were *se*-gene positive and carried *sec*, *sed*, and a *sea*–*sed* gene profile, respectively. The second most common AMR pattern was oxacillin resistance (OXA), which was observed in two *S. aureus* isolates, of which one isolate carried the *sec* gene. The remaining eleven AMR patterns were detected in a single, distinct *S. aureus* isolate. Notably, four *S. aureus* isolates exhibited a MRSA phenotype (5.4%; 4/74), as indicated by oxacillin resistance (MIC > 4 μg/mL) and confirmed by PCR detection of the *mecA* gene. One MRSA isolate, showing an antimicrobial resistance profile of OXA–CX, was recovered from the personal-use student computer group. The remaining three MRSA isolates exhibited a MDR phenotype (4.1%; 3/74). One MDR-MRSA isolate, with a resistance profile of CLI–ERY–FUS–LIN–NOV–OXA–PEN, was recovered from the public-use staff computer group. The other two MDR-MRSA isolates, exhibiting resistance profiles of CLI–ERY–NOV–OXA–PEN–TIG and CEF–CHL–CIP–CLI–ERY–GEN–LEV–LIN–MOX–NOV–OXA–PEN–TIG–TRI, were recovered from the personal-use student computer group. Interestingly, among the 52 *S. aureus* isolates that were phenotypically susceptible to all tested antimicrobial agents, 16.2% (12/74) carried *se* genes. The most frequently detected *se* gene in these susceptible isolates was *sec* (6 isolates), followed by *sea* (3 isolates), *sem* (2 isolates), and *seq* (1 isolate).

### 3.5. Association of Antimicrobial Resistance Patterns with DEC-Associated Virulence Genes in E. coli

The results showed that all six *E. coli* isolates were resistant to at least one of the tested antimicrobial agents (100%; 6/6), as shown in Table 6. Among these resistant isolates, three (50%) carried a single DEC-associated virulence gene profile, identified as *astA*. A total of four AMR patterns were observed in this study. Three *E. coli* isolates (50%) were classified as exhibiting a MDR phenotype. Two distinct MDR patterns were identified. The AMP–KAN–STR pattern was detected in two *E. coli* isolates, one recovered from the public-use staff computer group and the other from the personal-use student computer group; among these, one isolate harbored the *astA* gene. The second MDR pattern, AMP–GEN–KAN–STR, was identified in a single isolate recovered from the public-use staff computer group, in which no DEC-associated virulence genes were detected.

## 4. Discussion

In the present day, computers and their peripheral devices, particularly keyboards and mice, are widely used across occupational, educational, and public environments worldwide [25]. Because these devices are frequently touched during daily activities, they represent common contact surfaces that can accumulate microorganisms introduced through repeated hand contact and environmental exposure. Previous studies have demonstrated that computer keyboards and mice are often contaminated with a variety of bacteria, reflecting inadequate surface hygiene and inconsistent hand-washing practices among users [26]. Such contamination is of concern, as frequent human–device interaction may facilitate indirect microbial transmission, especially in shared or multi-user settings. This risk may be further amplified in the context of AMR, which has emerged as a major global public health challenge, as contaminated high-touch surfaces may contribute to the dissemination of resistant bacteria if awareness and hygiene practices are insufficient [27].

In this study, we investigated the prevalence of *S. aureus* and *E. coli* on computer devices, specifically the surface areas of keyboards and mice, used by staff and students at a university in Northern Thailand. In addition, the distribution of virulence-associated genes and antimicrobial resistance profiles, including MDR phenotypes, was evaluated.

The results demonstrated that the overall prevalence of *S. aureus* recovered from computer devices was 18.5%. This finding is comparable to previously reported prevalence rates of 17.4% in Iran [28] 18.64% in Iraq [2], and 19.5% in Saudi Arabia [29], where *S. aureus* was isolated from computer keyboards and mice. In contrast, several studies from other countries have reported substantially lower prevalence rates of *S. aureus* contamination on computer devices, including 3.22% in India [13], 3.57% in South Korea [30], 4% in Slovakia [1], 5% in Ghana [31], 6.97% in Saudi Arabia [32], and 10.39% in Iraq [33]. Conversely, higher prevalence rates than those observed in the present study have been documented in Ethiopia (23.6%) [12], Nigeria (39%) [34], and Libya (96.42%) [35].

With respect to *E. coli*, the overall prevalence detected in this study was relatively low (1.5%). This finding is consistent with previous reports from India (2.19%) [13] and Ghana (2.5%) [31]. However, higher *E. coli* prevalence rates have been reported in studies conducted in other regions, including Nigeria (7%) [34], Libya (8.33% and 14.28%) [35,36], Ethiopia (8.8%) [12], and Iraq (29.4%) [33]. The observed variation in the prevalence of *S. aureus* and *E. coli* across studies may reflect differences in hygiene practices, frequency of device sharing, environmental conditions, and sampling strategies among study settings.

Notably, the highest prevalence of *S. aureus* was observed in the personal-use student computer group (29%), which was significantly higher than that in the other groups (*p* < 0.0001). This elevated prevalence may be attributed to frequent and prolonged use of personal computer devices by students, resulting in repeated direct hand contact with high-touch surfaces and facilitating the transfer of skin-associated bacteria, including *S. aureus*, as previously reported for computer keyboards and related peripherals [26]. These findings may also reflect differences between user populations, as personal-use student computers are typically handled by individual users with frequent daily contact, whereas public-use computers are shared among multiple individuals with varying usage patterns and hygiene behaviors. However, detailed demographic characteristics of users could not be determined due to the anonymized study design and informed consent requirements. In contrast, *E. coli* was most frequently detected in the public-use staff computer group (4%); however, this difference was not statistically significant and may reflect incidental environmental or hygiene-related contamination, as fecal indicator bacteria such as *E. coli* have been identified on frequently touched surfaces in community and workplace environments and are associated with hygiene practices and environmental reservoirs rather than persistent colonization [37].

Staphylococcal enterotoxin genes in *S. aureus* isolates were evaluated to assess the pathogenic potential of circulating *S. aureus* strains. In the present study, 24.3% of *S. aureus* isolates were positive for at least one *se* gene, indicating the presence of potentially pathogenic phenotypes. The prevalence of *se*-gene-positive *S. aureus* observed in this study was higher than that reported by Sezer et al. [38], who documented a 17% prevalence among isolates recovered from food-contacting surfaces, utensils, and equipment. However, the se-gene positivity rate in the present study was lower than that reported in our previous investigation, in which 61.2% of *S. aureus* isolates from mobile phones harbored one or more se genes [19]. In this study, the four most frequently detected *se* genes were *sec* (13.5%), *sea* (5.4%), *sed*/*sem* (2.7%), and *seq* (1.4%). By contrast, our earlier study identified *sea* (32.7%), *sec* (20.4%), *seb* (10.2%), and *sem* (8.2%) as the predominant *se* genes, indicating notable variation in *se*-gene distribution between studies. Similar diversity in *se*-gene prevalence among *S. aureus* isolates from non-food and clinical-related samples has been reported previously. For example, Hamdan-Partida et al. [39] reported high frequencies of *seb* (57.8%) and *sea* (36.8%) in *S. aureus* isolates from mobile phones, while Sezer et al. [38] detected *sea* (84.38%), *sec* (43.75%), and *seb*/*sed* (3.13%) in isolates from food-contacting surfaces and equipment. In addition, Tasanapak et al. [40] reported lower detection rates of *sed* (3%) and *sec* (2%) among *S. aureus* recovered from kitchen equipment surfaces. Collectively, these findings suggest that variation in *se*-gene prevalence and profiles may be influenced by differences in sample sources, patterns of human contact, hygiene practices, and environmental conditions, supporting the role of high-touch surfaces as reservoirs for enterotoxigenic *S. aureus* strains [19,39]

DEC pathotypes are commonly characterized by the presence of specific virulence marker genes, including *aggR*, *stx1*, *stx2*, *astA*, *estp*, *esth*, *elt*, *bfpA*, *eae*, and *invE* [9]. In the present study, 3 (50%) of the six *E. coli* isolates were positive for a single DEC-associated virulence gene. This DEC-positive rate was higher than the prevalence reported by Arif et al. [41], who documented a DEC positivity of 16.67% among *E. coli* isolates recovered from public high-touch surfaces, such as ATM keyboards, public transport grab rails, elevator buttons, and restroom fixtures. In contrast, a substantially higher prevalence of DEC-positive *E. coli* (81%) was reported by Rakhalaru et al. [42] among isolates obtained from kitchen cloths and toilet surface samples. These differences suggest that the occurrence of DEC-associated virulence genes in *E. coli* recovered from non-food surfaces may vary according to surface type, intensity of human contact, and hygiene conditions within each environment [41,42]. In addition, the present study identified *astA* as the most frequently detected DEC-associated virulence gene, occurring in 50% (3/6) of the *E. coli* isolates. This prevalence was higher than that reported by Omar et al. [43], who documented a prevalence of 42.27% (123/291) among *E. coli* recovered from environmental water samples, but lower than the prevalence observed by Otokunefor et al. [44], who reported *astA* in 100% (9/9) of *E. coli* isolates obtained from Nigerian currency. Collectively, these findings indicate that the distribution of *astA*-positive *E. coli* varies across non-food environmental sources, likely reflecting differences in contamination pathways and human contact patterns.

This study investigated the AMR profiles of *S. aureus* strains recovered from computer devices used by staff and students in a university setting, contributing to ongoing surveillance of AMR as an emerging public health concern. Overall, the prevalence of resistance among the tested *S. aureus* isolates was low, with resistance rates of 1.4% for ciprofloxacin, 1.4% for chloramphenicol, 6.8% for erythromycin, and 1.4% for gentamicin. These findings are consistent with a study conducted in Iran, which reported similarly low resistance rates for ciprofloxacin (0%), chloramphenicol (6.7%), erythromycin (0%), and gentamicin (0%) among *S. aureus* isolates recovered from computer keyboards [45]. In contrast, higher resistance rates have been reported in studies from Ethiopia [12] and Ghana [31], suggesting that regional differences in antimicrobial usage practices, infection control measures, and hygiene behaviors may influence the observed AMR patterns. In the present study, all *S. aureus* isolates were susceptible to vancomycin. Although resistance was initially suggested by the disk diffusion assay, confirmation using the broth microdilution MIC method demonstrated that all isolates exhibited a susceptible phenotype. This result is consistent with findings from Iran, where vancomycin resistance was not detected among *S. aureus* isolates recovered from computer keyboards [45], and from Ethiopia [12], where vancomycin resistance was likewise absent among *S. aureus* isolates from non-clinical contact surfaces. This uniform susceptibility may reflect the restricted use of vancomycin, which is largely confined to clinical settings, thereby limiting selective pressure for the emergence of vancomycin resistance in non-clinical environments [46].

The prevalence of MRSA and MDR *S. aureus* in this study was 5.4% and 4.1%, respectively. The MDR prevalence was comparable to that reported by Bazgir et al. [45], who observed an MDR rate of 6.66%, although no MRSA isolates were detected in their study. In contrast, substantially higher MRSA and MDR prevalence rates have been documented in Ethiopia (15% MRSA and 87.5% MDR) [12] and Ghana (54.55% MDR) [31], highlighting considerable geographic variation in resistance patterns among *S. aureus* isolated from non-clinical environments.

Notably, three MRSA isolates identified in the present study exhibited MDR phenotypes, as shown in Table 5. All three isolates were negative for *se* genes, with one isolate recovered from a public-use staff computer and two from personal-use student computers. This observation suggests that antimicrobial resistance and enterotoxin gene carriage may occur independently in environmental *S. aureus* strains, as previously reported in studies indicating that resistance determinants and virulence factors do not necessarily co-occur in non-clinical isolates [12,45].

The AMR profiles of six *E. coli* isolates recovered from computer devices were evaluated in the present study. Resistance was observed against five of the tested antimicrobials, including gentamicin (16.7%), streptomycin (83.3%), kanamycin (50.0%), ampicillin (100%), and meropenem (16.7%). Notably, all *E. coli* isolates exhibited resistance to ampicillin, a finding consistent with previous reports from Ethiopia [12] and Ghana [31], where 100% ampicillin resistance was also observed among *E. coli* isolated from computer keyboards. Similar patterns of universal ampicillin resistance have been reported in studies involving non-food contact surfaces, including kitchen cloths and toilet surfaces in South Africa [42] and common public touch surfaces in Bangladesh [41], suggesting widespread selective pressure for ampicillin resistance in community and environmental settings. In contrast, low resistance rates were observed for chloramphenicol (0%) and gentamicin (16.7%) in the present study. These findings are comparable to those reported by Bazgir et al. [45], who documented complete susceptibility to both chloramphenicol and gentamicin among *E. coli* isolates recovered from computer keyboards. However, higher resistance rates to one or both of these agents have been reported elsewhere, including studies from Ethiopia (67% chloramphenicol and 33% gentamicin) [12], Ghana (75% chloramphenicol and 31.3% gentamicin) [31], and Bangladesh (8.3% for both agents) [41], indicating geographic variation in resistance profiles that may reflect differences in antimicrobial usage practices and environmental exposure.

MDR *E. coli* was detected in 50% (3/6) of the isolates in the present study, a prevalence higher than that reported in Iran (0%) [45] and South Africa (6.1%) [42]. Nevertheless, higher MDR prevalence rates have been documented in Ghana (62.5%) [31], Ethiopia (83.3%) [12], and Bangladesh (83.3%) [41], underscoring substantial regional differences in MDR *E. coli* occurrence on non-clinical contact surfaces. Two MDR resistance patterns—AMP–KAN–STR and AMP–GEN–KAN–STR—were identified in this study. All MDR *E. coli* isolates were recovered from computer devices used by staff (two isolates) and students (one isolate), and only one MDR isolate, recovered from a public-use staff computer, harbored the *astA* virulence gene. This observation suggests that multidrug resistance and DEC-associated virulence determinants may not consistently co-occur in *E. coli* strains recovered from non-food, non-clinical surfaces, as previously reported in environmental AMR surveillance studies [41,42].

This study has several limitations that should be acknowledged. First, the investigation was conducted at a single university in northern Thailand; therefore, the findings may not be fully representative of other academic institutions or geographic regions. Second, although the study provides initial insight into the prevalence, virulence characteristics, and AMR profiles of *S. aureus* and *E. coli*, a larger sample size across all target groups would improve the robustness and generalizability of the prevalence estimates. In addition, relevant metadata related to device characteristics and usage, such as device age, cleaning frequency, and usage frequency, were not collected in this study. Consequently, multivariable analyses (e.g., logistic regression) to evaluate potential risk factors could not be performed. Third, the study relied on phenotypic antimicrobial susceptibility testing and targeted PCR assays; the absence of high-throughput molecular approaches, such as whole-genome sequencing, limited comprehensive characterization of resistance mechanisms, virulence determinants, and clonal relatedness. In addition, meropenem-resistant *E. coli* isolates were not further screened for carbapenem-resistance genes. Therefore, the underlying genetic mechanisms responsible for the observed carbapenem resistance could not be determined. Finally, future studies incorporating multi-site sampling, larger populations, and advanced genomic analyses would provide a more detailed understanding of pathogen transmission dynamics and AMR dissemination on computer devices in academic environments.

## 5. Conclusions

This study demonstrates that computer devices used by staff and students in a university setting can harbor *S. aureus* and *E. coli*, including isolates carrying virulence-associated genes and antimicrobial resistance traits. Although the overall prevalence of antimicrobial-resistant strains, including MRSA and multidrug-resistant isolates, was relatively low, their detection highlights the potential role of frequently handled computer devices as occasional reservoirs of bacteria with public health relevance. The presence of *se* genes and DEC-associated virulence genes further underscores the importance of monitoring microbial contamination on shared equipment in academic environments. These findings support the need for improved hygiene practices, routine cleaning of computer devices, and awareness of antimicrobial resistance risks in non-clinical institutional settings. Continued surveillance may contribute to early detection and prevention strategies aimed at reducing microbial transmission within university communities.

## Figures and Tables

**Table 1:** Primer sequences used for PCR amplification of *Staphylococcus aureus* and *Escherichia coli* isolates in this study.

Gene	Primer Sequences (5′–3′)	Annealing Temperature (°C)	Amplified Products (bp)	References
*femA* ^a^	F: TAC GCA GCA TAT ACC GCA CT	54	300	[17]
	R: TAC GCA GCA TAT ACC GCA CT			
*mecA* ^b^	F: AAA ATC GAT GGT AAA GGT TGG C	51	533	[19]
	R: AGT TCT GCA GTA CCG GAT TTG C			
*mecC* ^b^	F: GCT CCT AAT GCT AAT GCA	51	304	[19]
	R: TAA GCA ATA ATG ACT ACC			
*sea* ^c^	F: ACG ATC AAT TTT TAC AGC	44.5	544	[19]
	R: TGC ATG TTT TCA GAG TTA ATC			
*seb* ^c^	F: ATT CTA TTA AGG ACA CTA AGT TAG GGGA	44.5	404	[19]
	R: ATC CCG TTT CAT AAG GCG AGT			
*sec* ^c^	F: GAC ATA AAA GCT AGG AAT TT	44.5	257	[19]
	R: AAA TCG GAT TAA CAT TAT CCA			
*sed* ^c^	F: CAA ATA TAT TGA TAT AAT GA	44.5	30	[19]
	R: AGT AAA AAA GAG TAA TGC AA			
*sej* ^c^	F: CAC CAG AAC TGT TGT TCT GCTAG	55	114	[19]
	R: CTG AAT TTT ACC ATC AAA GGTAC			
*sel* ^c^	F: TGG ACA TAA CGG CAC TAA AA	52	145	[19]
	R: TTG GTA RCC CAT CAT CTC CT			
*sem* ^c^	F: AGT TTG TGT AAG AAG TCA AGT GTA GA	52	178	[19]
	R: ATC TTT AAA TTC AGC AGA TAT TCC ATC TAA			
*seq* ^c^	F: ATA CCT ATT AAT CTC TGG GTC AAT G	52	222	[19]
	R: AAT GGA AAG TAA TTT TTC CTTTG			
*ser* ^c^	F: TCC CAT TCC TTA TTT AGA ATACA	52	440	[19]
	R: GGA TAT TCC AAA CAC ATC TGAC			
*uidA* ^d^	F: TAT GGA ATT TCG CCG ATT TT	55	166	[18]
	R: TGT TTG CCT CCC TGC TGC GG			
*aggR* ^e^	F: GTA TAC ACA AAA GAA GGA AGC	55	254	[18]
	R: ACA GAA TCG TCA GCA TCA GC			
*astA* ^e^	F: CCA TCA ACA CAG TAT ATC CG	55	101	[18]
	R: ACG GCT TTG TAG TCC TTC CA			
*bfpA* ^e^	F: AAT GGT GCT TGC GCT TGC TGC	61	324	[18]
	R: GCC GCT TTA TCC AAC CTG GTA			
*eae* ^e^	F: CCC GAA TTC GGC ACA AGC ATA AGC	61	881	[18]
	R: CCC GGA TCC GTC TCG CCA GTA TTC G			
*esth* ^e^	F: TTC ACC TTT CCC TCA GGA TG	57	172	[18]
	R: ATA GCA CCC GGT ACA AGC AG			
*elt* ^e^	F: AAC GTT CCG GAG GTC TTA TG	57	511	[18]
	R: CAA CCT TGT GGT GCA TGA TC			
*estp* ^e^	F: ACT GAA TCA CTT GAC TCT TCA	55	120	[18]
	R: TCA CAG CAG TAA ATG TGT TGT			
*invE* ^e^	F: GCA GGA GCA GAT CTT GAA C	55	208	[18]
	R: GAA AGG CAC GAG TGA CTT TC			
*stx* ^e^	F: AGT TAT GTG GTG GCG AAG G	55	347	[18]
	R: CAC CAG ACA ATG TAA CCG C			
*stx2* ^e^	F: TTC GGT ATC CTA TTC CCG G	55	589	[18]
	R: CGT CAT CGT ATA CAC AGG AG			

Note: Lowercase letters (a–e) correspond to the following categories: (a) genetic markers for *S. aureus* identification, (b) MRSA identification, (c) *S. aureus* virulence genes, (d) genetic markers for *E. coli* identification, and (e) *E. coli* virulence genes. *femA*, factor essential for methicillin resistance A; *mecA*, methicillin resistance gene A; *mecC*, methicillin resistance gene C; *sea*, staphylococcal enterotoxin A; *seb*, staphylococcal enterotoxin B; *sec*, staphylococcal enterotoxin C; *sed*, staphylococcal enterotoxin D; *sej*, staphylococcal enterotoxin J; *sel*, staphylococcal enterotoxin L; *sem*, staphylococcal enterotoxin M; *seq*, staphylococcal enterotoxin Q; *ser*, staphylococcal enterotoxin R. *uidA*, β-glucuronidase gene; *aggR*, transcriptional activator of enteroaggregative *E. coli*; *astA*, enteroaggregative heat-stable toxin 1 gene; *bfpA*, bundle-forming pilus gene; *eae*, intimin gene; *esth*, heat-stable enterotoxin (STh) gene; *elt*, heat-labile enterotoxin (LT) gene; *estp*, heat-stable enterotoxin (STp) gene; *invE*, invasion-associated gene; *stx1*, Shiga toxin 1 gene; *stx2*, Shiga toxin 2 gene.

**Table 2:** The prevalence of *Staphylococcus aureus* and *Escherichia coli* isolated from computer devices across different sample groups.

Sample Groups	Number of Samples	*S. aureus* Positive Sample; n (%)	*E. coli* Positive Sample; *n* (%)	*p*-Values
Public-use staff computers	100	20 (20)	4 (4)	<0.0001 ^a^
Personal-use staff computers	100	21 (21)	0	0.1072 ^b^
Public-use student computers	100	4 (4)	1 (1)	
Personal-use student computers	100	29 (29)	1 (1)	
Total	400	74 (18.5)	6 (1.5)	

*p*-values were calculated using the chi-square test for comparisons of (^a^) *S. aureus* prevalence distribution and (^b^) *E. coli* prevalence distribution among sample groups.

**Table 3:** Distribution of *Staphylococcus aureus* isolates harboring enterotoxin genes from computer keyboards and computer mice among four different sample groups.

Sample Group	No. of *S. aureus* Isolates	No. (%) of *S. aureus* Harboring *se* Gene	No. (%) of *S. aureus* Harbored								
			*sea*	*seb*	*sec*	*sed*	*sej*	*sem*	*sel*	*seq*	*ser*
Public-use staff computers	20	2 (10)	0	0	1 (5)	1 (5)	0	0	0	0	0
Personal-use staff computers	21	8 (38.1)	1 * (4.8)	0	6 (28.6)	1 * (4.8)	0	0	0	1 (4.8)	0
Public-use student computers	4	1(25)	1 (25)	0	0	0	0	0	0	0	0
Personal-use student computers	29	7 (24.1)	2 (6.9)	0	3 (10.3)	0	0	2 (6.9)	0	0	0
Total	74	18 (24.3)	4 (5.4)	0	10 (13.5)	2 (2.7)	0	2 (2.7)	0	1 (1.4)	0
*p*-value	0.2219										

*p*-values were calculated using the chi-square test to compare the distribution of *se* gene-positive and *se* gene-negative *S. aureus* isolates among sample groups. * Single *S. aureus* isolates harbored the *sea–sed* gene profile. *sea*, *seb*, *sec*, *sed*, *sej*, *sem*, *sel*, *seq*, and *ser* represent staphylococcal enterotoxin genes A, B, C, D, J, M, L, Q, and R, respectively.

**Table 4:** Distribution of *Escherichia coli* isolates harboring DEC-associated virulence genes from computer keyboards and computer mice among four different sample groups.

Sample Group	No. of *E. coli* Isolates	No. (%) of *E. coli* Harboring Virulence Gene	No. (%) of *E. coli* Harbored									
			*aggR*	*astA*	*bfpA*	*eae*	*esth*	*elt*	*estp*	*invE*	*stx*	*stx2*
Public-use staff computers	4	2 (50)	0	2 (50)	0	0	0	0	0	0	0	0
Personal-use staff computers	0	0	0	0	0	0	0	0	0	0	0	0
Public-use student computers	1	1 (100)	0	1 (100)	0	0	0	0	0	0	0	0
Personal-use student computers	1	0	0	0	0	0	0	0	0	0	0	0
Total	6	3 (50)	0	3 (50)	0	0	0	0	0	0	0	0
*p*-value	0.3149											

*p*-values were calculated using the chi-square test to compare the distribution of virulence gene-positive and virulence gene-negative *E. coli* isolates among sample groups.

**Table 5:** Profiles of antimicrobial resistance and enterotoxin (*se*) genes in *Staphylococcus aureus* isolates.

Antimicrobial Resistance Profiles ^a^	No. of *S. aureus* Isolates	No. of *se* Gene-Positive Isolates	No. of Isolates Harboring Staphylococcal Enterotoxin Genes					
			*sea*	*sec*	*sed*	*sem*	*seq*	*sea-sed*
CLI	9	3	0	1	1	0	0	1
OXA	2	1	0	1	0	0	0	0
CEF	1	0	0	0	0	0	0	0
ERY	1	1	0	1	0	0	0	0
LIN	1	1	0	1	0	0	0	0
OXA-CX ^b^	1 ^c^	0	0	0	0	0	0	0
CLI-ERY	1	0	0	0	0	0	0	0
CLI-MOX	1	0	0	0	0	0	0	0
CLI-TRI	1	0	0	0	0	0	0	0
PEN-LEV	1	0	0	0	0	0	0	0
**CLI-ERY-NOV-OXA-PEN-TIG** ^b^	1 ^c^	0	0	0	0	0	0	0
**CLI-ERY-FUS-LIN-NOV-OXA-PEN** ^b^	1 ^d^	0	0	0	0	0	0	0
**CEF-CHL-CIP-CLI-ERY-GEN-LEV-LIN-MOX-NOV-OXA-PEN-TIG-TRI** ^b^	1 ^c^	0	0	0	0	0	0	0
Resistance	22	6	0	4	1	0	0	1
Susceptible	52	12	3	6	0	2	1	0
Total	74	18	3	10	1	2	1	1

^a^: Antimicrobial agents tested—CEF, cefoxitin; CHL, chloramphenicol; CIP, ciprofloxacin; CLI, clindamycin; ERY, erythromycin; FUS, fusidic acid; GEN, gentamicin; LEV, levofloxacin; LIN, linezolid; MOX, moxifloxacin; NOV, novobiocin; OXA, oxacillin; PEN, penicillin G; TGC, tigecycline; TRI, trimethoprim/sulfamethoxazole; ^b^: MRSA-positive strains confirmed by detection of the *mecA* gene using PCR. ^c^: Isolate recovered from personal-use student computer group; ^d^: Isolate recovered from public-use staff computer group. The bold letter refers to MDR of *S. aureus* isolates.

**Table 6:** Profiles of antimicrobial resistance and pathotype-associated virulence genes in *Escherichia coli* isolates.

Antimicrobial Resistance Profiles ^a^	No. of *E. coli* Isolates	No. of Virulence Gene Positive Isolates	No. of Isolates Harboring Pathotype-Associated Virulence Genes
			*astA*
AMP-STR	2	1	1
AMP-MER	1	1	1
**AMP-KAN-STR**	2 (1 ^b^ + 1 ^c^)	1 ^b^	1
**AMP-GEN-KAN-STR**	1 ^b^	0	0
Resistance	6	3	3
Susceptible	0	0	0
Total	6	3	3

^a^: Antimicrobial agents tested: AMP, Ampicillin; MER, Meropenem; GEN, Gentamicin; KAN, Kanamycin; STR, Streptomycin. ^b^: Isolate recovered from public-use staff computer group; ^c^: Isolate recovered from personal-use student computer group. The bold letter refers to MDR of *E. coli* isolates.

## Data Availability

The data supporting the findings of this study are provided within the manuscript. Additional information is available from the corresponding authors upon reasonable request.
